# Longitudinal Fecal Short-Chain Fatty Acid Trajectories in Preterm Infants with Early-Onset Neonatal Sepsis: A Pilot Study

**DOI:** 10.3390/life15121943

**Published:** 2025-12-18

**Authors:** Evgenii Kukaev, Olga Krogh-Jensen, Natalia Starodubtseva, Alisa Tokareva, Irina Nikitina, Anna Lenyushkina, Vladimir Frankevich, Gennady Sukhikh

**Affiliations:** 1 V.I. Kulakov National Medical Research Center for Obstetrics Gynecology and Perinatology, Ministry of Healthcare of Russian Federation, 117997 Moscow, Russia; o_krogh@oparina4.ru (O.K.-J.); n_starodubtseva@oparina4.ru (N.S.); a_tokareva@oparina4.ru (A.T.); i_nikitina@oparina4.ru (I.N.); a_lenyushkina@oparina4.ru (A.L.); v_frankevich@oparina4.ru (V.F.); g_sukhikh@oparina4.ru (G.S.); 2 V. L. Talrose Institute for Energy Problems of Chemical Physics, N. N. Semenov Federal Research Center for Chemical Physics, Russian Academy of Sciences, 119334 Moscow, Russia; 3 Moscow Center for Advanced Studies, 123592 Moscow, Russia; 4 Neonatal Department of the Institute of Children’s Health, Federal State Autonomous Educational Institution of Higher Education I.M. Sechenov First Moscow State Medical University of the Ministry of Health of the Russian Federation, 119991 Moscow, Russia; 5 Laboratory of Translational Medicine, Siberian State Medical University, 634050 Tomsk, Russia; 6 Department of Obstetrics, Gynecology, Perinatology and Reproductology, Institute of Professional Education, Federal State Autonomous Educational Institution of Higher Education I.M. Sechenov First Moscow State Medical University of the Ministry of Health of the Russian Federation, 119991 Moscow, Russia

**Keywords:** early-onset neonatal sepsis (EONS), preterm infants, short-chain fatty acids (SCFAs), gas chromatography–mass spectrometry (GC–MS), gut microbial metabolism, ecrotizing enterocolitis (NEC), intestinal vulnerability, longitudinal analysis, neonatal biomarkers

## Abstract

Background: Early-onset neonatal sepsis (EONS), defined as systemic infection occurring within the first 72 hours of life, remains a major cause of morbidity and mortality in preterm infants. Increasing evidence indicates that the gut may play an active role in systemic inflammation, yet the temporal behavior of fecal short-chain fatty acids (SCFAs) during EONS has not been characterized. SCFAs and branched-chain fatty acids (BCFAs) are key microbial metabolites involved in epithelial maturation and immune regulation and may provide a non-invasive window into early inflammatory vulnerability. Methods: This pilot prospective longitudinal cohort study enrolled 49 preterm infants (≤32 weeks’ gestation) originally identified as at high risk for necrotizing enterocolitis (NEC) and subsequently stratified into EONS and non-sepsis groups. Serial stool samples were collected at predefined timepoints (TPs; TP1 ≈ 3 days of life [DoL], TP2 ≈ 7 DoL, TP3 ≈ 14 DoL, TP4 ≈ 21 DoL, and TP5 ≈ 28 DoL). Samples were analyzed using gas chromatography–mass spectrometry (GC–MS) to quantify a panel of 12 SCFAs, including BCFAs and medium-chain fatty acids (MCFAs). Both absolute concentrations and relative fractions were evaluated, with emphasis on ratio-based metrics (e.g., acetic/propionic acid ratio) and timepoint-specific group contrasts, complemented by partial least squares discriminant analysis (PLS–DA). Results: At the earliest sampling window (TP1), infants with EONS exhibited distinct early changes in SCFA composition, including a significantly lower median relative fraction of acetic acid (86.6% vs. 94.5% in non-sepsis), while several non-acetate components—including propionic, valeric, and branched-chain acids—were relatively enriched. Acetate-to-non-acetate ratios were markedly reduced in EONS (e.g., acetic/propionic and acetic/isobutyric ratios), indicating an early shift away from acetate dominance. PLS–DA at TP1 demonstrated partial separation between groups, with acetic-acid depletion and non-acetate enrichment among the strongest contributors to discrimination. By later TPs, these early differences narrowed to a small subset of BCFA-related ratios and largely attenuated by the end of the first month. Conclusions: In this pilot cohort of preterm infants, EONS was associated with early, structured alterations in fecal SCFA profiles, characterized by reduced acetic-acid dominance and relative enrichment of non-acetate acids. Dynamic, ratio-based assessment proved more informative than absolute concentrations alone, revealing transient intestinal metabolic signatures accompanying systemic infection. These findings provide the first longitudinal evidence of gut metabolic involvement in EONS and lay the groundwork for larger, multi-center studies integrating SCFA trajectories with microbiome and immune profiling to refine early risk stratification for systemic infection in high-risk neonatal populations.

## 1. Introduction

Neonatal morbidity and mortality remain unacceptably high worldwide, particularly among preterm infants, in whom immature organ systems and a fragile immune response predispose to life-threatening complications. Early-onset neonatal sepsis (EONS) and necrotizing enterocolitis (NEC) are among the most severe and socially significant disorders in this population, contributing substantially to both acute mortality and long-term neurodevelopmental and somatic sequelae [1,2,3,4,5,6]. Both conditions arise at the interface between an immature host and a rapidly changing microbial environment, and there is growing interest in how gut-derived metabolites, particularly short-chain fatty acids (SCFAs), may modulate susceptibility and clinical trajectories [7,8].

EONS, typically defined as sepsis occurring within the first 72 hours of life [6], remains a major cause of death and severe morbidity in preterm and preterm neonates. Its pathogenesis reflects a complex interplay between vertical and early postnatal microbial exposures, inadequate innate immune responses, and impaired epithelial and endothelial barrier function [9]. Recent work increasingly points to the gut as an important reservoir and signaling hub in neonatal sepsis: dysbiosis, delayed colonization by beneficial taxa, enrichment of opportunistic pathogens, and altered microbial metabolic output are all associated with sepsis risk [7,10]. Among microbial metabolites, SCFAs and related fatty acids have attracted particular attention in the context of systemic inflammation and septic shock [11,12,13]. However, most available data come from cross-sectional measurements or from non-intestinal compartments, and, to date, the temporal dynamics of fecal SCFAs during EONS have not been comprehensively characterized.

NEC, in turn, remains one of the most feared gastrointestinal emergencies of the neonatal period, with multifactorial pathogenesis involving prematurity, enteral feeding, microbial imbalances, and exaggerated inflammatory responses [1,2,3]. Disturbances in host–microbiota interactions and barrier integrity are central to NEC development, and SCFAs have been proposed as key mediators in this triad [14,15,16]. Early clinical stages of NEC suspicion (Bell stage I) are now recognized as a distinct and clinically relevant phenotype rather than mere false alarms [17,18,19]. Metabolomic and microbiome studies indicate that infants in this “NEC-risk” state already exhibit altered microbial composition and metabolic signatures, even when the disease does not progress to overt intestinal necrosis [8,17]. Importantly, some of these early intestinal disturbances may reflect broader systemic inflammatory vulnerability, conceptually linking NEC-risk states and sepsis-prone phenotypes without equating the two conditions [20].

SCFAs (including acetic, propionic, and butyric acids) and related branched chain fatty acids (BCFAs) exert multiple homeostatic functions at the mucosal interface. They provide energy substrates for colonocytes, regulate epithelial proliferation and differentiation, influence tight-junction protein expression, and modulate cytokine production and immune-cell activation [14,15,21,22,23,24]. BCFAs such as isobutyric and isovaleric acids, which arise from proteolytic fermentation, offer additional insight into microbial metabolic activity in the immature gut [25,26]. Imbalances in these metabolites have been implicated in inflammatory bowel diseases and in microbiota disturbances in preterm infants [27,28,29,30], suggesting that SCFA patterns may serve as sensitive indicators of host–microbe dysregulation.

Despite this growing body of evidence, a critical gap remains in our understanding of how fecal SCFA profiles evolve over time in preterm infants who develop EONS. Existing studies in neonatal sepsis have largely focused on single timepoints, heterogeneous septic phenotypes, or systemic (plasma/serum) markers [11,12,13,31,32], providing limited insight into the intestinal metabolic trajectories that accompany early systemic infection. It is therefore unclear whether EONS is associated with reproducible, time-dependent shifts in the intestinal SCFA and BCFA pools, and whether such patterns might reflect either vulnerability, compensatory adaptation, or metabolic exhaustion of the gut ecosystem.

In this context, we designed a longitudinal cohort study originally motivated by the pathophysiology of NEC. However, during the enrolment period, the incidence of NEC in the targeted gestational window was unexpectedly low, making adequately powered NEC-focused analyses unfeasible [17,33,34]. At the same time, because NEC and EONS share key axes of gut–immune interaction—including dysbiosis, impaired epithelial barrier function, and early inflammatory signaling—the planned high-resolution, time-structured sampling framework remained biologically appropriate [6,7,8,10,35,36]. These circumstances provided an opportunity to focus the primary analyses on preterm infants with and without early-onset neonatal sepsis while preserving the original temporal design. The present study therefore aimed to characterize absolute and relative fecal SCFA concentrations across the first month of life in preterm neonates initially enrolled as at high risk for NEC but subsequently stratified by EONS status. By performing dynamic, timepoint-specific analyses rather than relying on single measurements, we sought to determine whether EONS is accompanied by distinct intestinal SCFA signatures. To our knowledge, this is the first longitudinal assessment of a comprehensive fecal SCFA and BCFA panel in the context of early-onset neonatal sepsis, laying groundwork for future studies that will extend this approach to dedicated NEC cohorts and refined risk phenotypes.

In addition, extending SCFA profiling across the entire first postnatal month is biologically and clinically justified. The neonatal gut undergoes its most rapid and nonlinear development during the first 2–4 weeks of life, a period characterized by intense microbial succession, maturation of mucosal immunity, and substantial shifts in microbial fermentation. Early-life antibiotic exposure, which is common in preterm infants, has been shown to destabilize the gut microbiome and alter its metabolic output for several weeks after birth, indicating that early systemic events may have prolonged intestinal consequences [37]. Dynamic changes in SCFA production during the first month have also been demonstrated experimentally: an in vitro fermentation study using preterm infant stool showed that the production of acetic, propionic, butyric, and branched-chain fatty acids evolves substantially across the first four weeks of life, reflecting continuous maturation of microbial functional capacity [9]. Similar longitudinal behavior has been observed for other fecal biomarkers in preterm infants; for example, fecal calprotectin exhibits high biological variability and marked week-to-week fluctuations during the first month of life [38,39]. Collectively, these findings support the rationale for a longitudinal design, as early-onset sepsis may influence not only immediate microbial activity but also the developmental trajectory of intestinal metabolic function during this critical maturation period.

## 2. Materials and Methods

### 2.1. Study Design

This prospective longitudinal cohort pilot study was conducted at the Neonatal Intensive Care Unit of the V.I. Kulakov National Medical Research Center for Obstetrics, Gynecology, and Perinatology from June 2024 to February 2025. Preterm neonates (gestational age ≤ 32 weeks) were enrolled; the study was approved by the local ethics committee (Protocol 04, 18 April 2024), and written informed consent was obtained from parents or legal guardians.

Eligible infants were admitted to the NICU with gestational age ≤ 32 weeks; exclusion criteria included severe congenital malformations, chromosomal abnormalities, inherited metabolic disorders, and hydrops fetalis. In addition, we collected stool samples in more mature neonates (GA > 32 weeks) if they developed signs suspicious for NEC (one or more signs: pronounced abdominal distension, pain on abdominal palpation, frequent regurgitation, blood in the stool, lack of intestinal peristalsis). The samples were taken on the day these signs appeared. NEC diagnosis was based on the modified Bell’s criteria [40] and assessed by two independent clinical experts (I.N., O.K.J.) before obtaining the results of SCFA testing. These more mature infants with suspected NEC (GA > 32 weeks) formed an ancillary descriptive cohort and were not included in the primary EONS vs. non-EONS statistical analyses.

Given real-world enrollment, the original NEC-centered plan was adapted to focus on early-onset neonatal sepsis (EONS) versus absence of sepsis while retaining an exploratory NEC-risk subgroup. Early-onset neonatal sepsis was defined as the presence of at least two clinical symptoms and at least two laboratory signs in the presence of, or as a result of, suspected or proven infection (positive culture, microscopy, or polymerase chain reaction) within the first 72 h of life, according to the criteria listed in the expert report on neonatal and pediatric sepsis by the European Medicines Agency [41]. Infants fulfilling these criteria comprised the EONS group; infants without clinical or laboratory evidence of sepsis during the observation windows comprised the non-EONS group. All cases of EONS were evaluated independently by two experts (I.N. and O.K.J.) before the results of SCFA testing became available. All neonates were followed up until discharge from the NICU.

Fecal sampling followed predefined postnatal windows centered at approximately day of life (DoL) 3, 7, 14, 21, and 28 (hereafter TP1–TP5); when multiple samples occurred within a window, the specimen closest to the window’s median timing was selected for primary analyses. To improve reproducibility, the effective sampling ranges were: TP1 (DoL 1–5), TP2 (DoL 6–11), TP3 (DoL 11–19), TP4 (DoL 17–26), and TP5 (DoL 26–30).

Notwithstanding this design, we additionally characterized, for descriptive and contextual purposes, a small ancillary cohort of infants who developed confirmed NEC (Bell II) and suspected NEC (Bell I) with gestational age > 32 weeks. These cases were reviewed under the same ethics approval and are presented solely as a post hoc, hypothesis-generating extension; they were not included in the primary EONS vs. non-sepsis longitudinal analyses.

### 2.2. Chemicals and Reagents

For the quantitative GC–MS analysis of SCFAs in feces, analytical-grade standards and reagents were obtained from reliable suppliers (Sigma-Aldrich, Saint Louis, MO, USA). The reference standards included acetic (AA, ≥99%), propionic (PA, ≥99%), butyric (BA, ≥99%), valeric (VA, ≥99%), isobutyric (iBA, ≥99%), isovaleric (iVA, ≥99%), 2-methylbutyric (2mBA, ≥99%), 2-methylvaleric (2mVA, ≥99%), 3-methylvaleric (3mVA, ≥99%), hexanoic (HA, ≥99%), and octanoic (OA, ≥99%) acids.

To improve quantification accuracy and correct for matrix effects, internal standards (IS) were used. AA-d_4_ (≥99.5%) served as the internal standard for acetic acid, while BA-1,2-^13^C_2_ (≥98%) was applied for other SCFAs, including PA, BA, VA, iBA, iVA, 2mBA, 2mVA, 3mVA, HA, and OA.

Milli-Q water was used for the preparation and dilution of calibration and working solutions, while 2.0 M hydrochloric acid (HCl) was employed for acidification prior to extraction. Methyl tert-butyl ether (MTBE) served as the solvent for liquid–liquid extraction (LLE) of SCFAs, ensuring efficient recovery and phase separation.

All reagents and consumables were handled in accordance with standard laboratory protocols and quality-control procedures to ensure the reliability and reproducibility of analytical results [42,43].

### 2.3. Sample Collection

Naturally excreted stool samples were collected on days 3, 7, 14, 21, and 28 of life, preferably before antibiotic therapy or the morning feeding. Samples were placed in sterile containers with inert plastic inserts to prevent contact with the diaper surface and minimize volatile loss. All samples were immediately frozen at −80 °C until analysis.

In infants who developed acute intestinal symptoms during hospitalization (e.g., cases later evaluated for suspected or confirmed NEC), stool samples were additionally collected within 24 h after symptom onset according to unit protocol. Enteral feeding was discontinued at the time of symptom appearance; therefore, the feeding characteristics reported in the Results correspond to the last 24 h of enteral nutrition prior to clinical deterioration. These cases were not part of the longitudinal comparison and are presented descriptively.

### 2.4. Sample Preparation

A 50–100 mg aliquot of native stool was weighed into 1.6–2.0 mL Eppendorf tubes. Milli-Q water (1000 µL per 100 mg stool) was added, and the exact dilution factor was determined gravimetrically. After GC–MS analysis, SCFA concentrations (µM) were recalculated to µmol per g of stool. Samples were vortexed, sonicated for 20 min at room temperature, vortexed again for 10 min, and centrifuged (5000 rpm, RT). Then 100 µL of supernatant was transferred to a 0.5 mL tube, 5 µL of internal standard solution (d_4_-acetic acid 10^3^ µM, ^13^C_2_-butyric acid 10^2^ µM) and 5 µL of 2 M HCl were added, followed by 200 µL of methyl tert-butyl ether (MTBE). After vortexing (10 min) and centrifugation (15,000 rpm, 5 min), the upper organic phase was transferred to an autosampler vial for GC–MS analysis.

The protocol was adapted from previously published methods, demonstrating the applicability of water as a surrogate matrix and MTBE extraction after acidification. Kim et al. (2022) [43] established a methodological basis validated across several bio-matrices, whereas our work [42] applied and tested it on clinical stool and plasma samples. In this study, the protocol was further modified for neonatal stool, to reduce matrix effects and improve reproducibility.

### 2.5. Calibration Standards and Quality Controls

Calibration standards were prepared in Milli-Q water, with sample preparation identical to that of the stool extracts, beginning with a 100 µL aqueous aliquot. Stock solutions of the analytes were prepared in acetonitrile at the following concentrations: AA at 10^6^ µM; PA, BA, and iBA acids at 10^5^ µM; and VA, iVA, 2mBA, 2mVA, 3mVA, HA, and OA at 10^4^ µM. These stock standards were stored at ≤+4 °C and serially diluted with water to create working solutions. The internal standards (IS) consisted of d_4_-AA (10^4^ µM, stored at ≤ −20 °C) and ^13^C_2_-BA (10 mg/mL, stored at ≤ −20 °C). A working internal standard (ISTD) solution containing 10^3^ µM d_4_-AA and 10^2^ µM ^13^C_2_-BA was prepared, with 5 µL added to each sample.

Seven-point calibration curves and four levels of quality-control (QC) standards were prepared in Milli-Q water, which was selected as a surrogate matrix based on previous validation data [42,43] and confirmed by standard-addition tests in this study. Calibration and QC samples underwent identical preparation steps to fecal extracts, starting from the stage of 100 µL aliquot acidification and MTBE extraction. For AA, calibration levels were 2, 4, 10, 20, 40, 100, and 160 µM, with an additional high-level point at 2000 µM when needed to account for elevated concentrations. For PA, BA and iBA, calibration levels were 0.2, 0.4, 1, 2, 4, 10, and 16 µM, with an optional extended level of 200 µM for high-concentration samples. For other acids (2mBA, iVA, VA, 2mVA, 3mVA, HA, OA), levels were 0.02, 0.04, 0.1, 0.2, 0.4, 1, and 1.6 µM, optionally extended to 20 µM.

Calibration curves were constructed by plotting the peak area ratio of each SCFA to its corresponding internal standard against the analyte concentration, followed by linear regression (Table 1). The linearity for each SCFA was confirmed by a coefficient of determination (R^2^) exceeding 0.995. The limit of detection (LOD) was calculated as 3.3×SD/b, where SD is the standard deviation of the Y-intercept and b is the slope of the regression curve. The limit of quantification (LOQ) was defined as 3×LOD. For several samples, the quantification was additionally verified using the standard addition method with four spiked concentration levels.

### 2.6. GC–MS Analysis

Analyses were performed using an Agilent 7890B/5977B GC–MS system (Agilent Technologies, Santa Clara, CA, USA) equipped with an HP-FFAP capillary column (30 m × 0.25 mm i.d., 0.25 µm film thickness; Agilent Technologies, Santa Clara, CA, USA). Helium was used as the carrier gas at a constant flow of 1.7 mL/min. The oven temperature was programmed as follows: initial temperature 60 °C (held for 1.5 min), ramped at 20 °C/min to 240 °C, and held for 7.5 min. Electron ionization (EI) at 70 eV was used with a solvent delay of 4 min. Injector, ion source, and quadrupole temperatures were maintained at 240 °C, 230 °C, and 150 °C, respectively.

A preliminary series of full-scan measurements was performed to determine retention times and identify characteristic fragment ions by spectral matching with the NIST17 library [44]. Subsequently, ion registration was carried out in SIM (Single Ion Monitoring) mode for quantitative determination of individual analytes (Table 1). Quantification was performed in Selected Ion Monitoring (SIM) mode by targeting characteristic fragment ions for each analyte. The ion at *m*/*z* 43 was monitored for isobutyric acid (iBA), while the ion at *m*/*z* 60 was used for the simultaneous detection of acetic, butyric, valeric, isovaleric, and 3-methylvaleric acids. Propionic, 2-methylbutyric, and 2-methylvaleric acids were quantified using the ion at *m*/*z* 74. For the IS, the ions *m*/*z* 46 and 63 were selected for the deuterated standard d_4_-acetic acid, and the ion *m*/*z* 62 was selected for ^13^C_2_-butyric acid. Compound identity was confirmed by retention time and spectral comparison with authentic standards (NIST17). Method performance (R^2^ > 0.995, LOD 0.5–1 µM, accuracy ±10 %, CV < 15 %) and extract stability at −80 °C up to 14 days were consistent with published data [42,43].

### 2.7. Statistical Analysis

All statistical analyses were performed in Python 3.13 using the packages pandas (data handling), NumPy (numerical operations), SciPy (non-parametric tests), scikit-learn (multivariate modeling), and matplotlib/seaborn (visualization).

Continuous variables are summarized as median (Q1; Q3), and categorical variables as *n* (%). Between-group comparisons for clinical characteristics were carried out using the Mann–Whitney *U* test for continuous variables and Fisher’s exact test for categorical variables. Statistical significance was defined as p<0.05; values with 0.05≤p<0.10 were interpreted as trends.

For fecal short-chain fatty acid (SCFA) analysis, both absolute concentrations and derived metrics were evaluated. Derived variables included:

- relative fractions, ${\mathrm{SCFA}}_{\mathrm{rel}}$ (percentage of total SCFAs);
- composite branched-chain indices, Branch and ${\mathrm{Branch}}_{\mathrm{rel}}$;
- pairwise ratios of metabolites, ${\mathrm{SCFA}}_{i}/{\mathrm{SCFA}}_{j}$.

For each analyte, timepoint-specific differences between the EONS and non-sepsis groups were assessed at TP1–TP5 (approximately 3, 7, 14, 21, and 28 days of life) using the Mann–Whitney *U* test. Infants evaluated for suspected or confirmed NEC formed a small exploratory subgroup; their SCFA profiles were summarized descriptively and were not included in inferential testing.

Multivariate analysis was performed using partial least squares–discriminant analysis (PLS–DA) implemented in scikit-learn. Variable Importance in Projection (VIP) scores were calculated for each metabolite and ratio; variables with VIP ≥1 were considered informative contributors to group separation. Temporal patterns were visualized using boxplots, PLS–DA score plots, and median trend curves across days of life, with *p*-values annotated on timepoint-specific plots where appropriate.

### 2.8. Ethical Approval

The study was conducted in accordance with the Declaration of Helsinki and approved by the local ethics committee (18 April 2024, # 04). Informed consent was obtained from parents or legal guardians of all participants.

## 3. Results

### 3.1. Participants

A total of N = 49 preterm infants were included in the study, of whom n = 18 were diagnosed with early-onset neonatal sepsis (EONS group) and n = 31 had no evidence of sepsis (non-sepsis group). Baseline demographic and clinical characteristics of the cohort are summarized in Table 2. Continuous variables are reported as median (Q1–Q3) and were compared using the Mann–Whitney *U* test; categorical variables are shown as *n* (%) and were compared using two-sided Fisher’s exact test, with p<0.05 considered significant. All infants underwent clinical and laboratory evaluation within the first 6 h after birth, and clinical suspicion of EONS in all affected neonates arose during this early evaluation period. Laboratory confirmation of EONS was established within the first 72 h of life in accordance with the predefined diagnostic criteria. All infants with EONS received empiric ampicillin–gentamicin therapy within the first 6 h of life, coinciding with initial clinical assessment; therefore, TP1 stool samples (≈3 days of life) were collected after both the onset of EONS and the initiation of antibiotic treatment. Most newborns in the non-EONS group (27/31) also received empirical antibacterial therapy beginning on the first day of life; in 10 of these infants treatment was discontinued after 48 h once EONS or pneumonia had been excluded.

Groups did not differ in gestational age at birth (31 [28.5–32] vs. 31.2 [29.3–32] weeks; p=0.10) or sex distribution (p=0.39). Birth weight was lower in EONS (1121 [840–1329] vs. 1440 [1092–1679] g; p=0.049). Apgar scores were lower in EONS at 1 min (p<0.001) and 5 min (p=0.03). Surfactant treatment and invasive mechanical ventilation were more frequent in EONS (p<0.001 and p=0.001, respectively). Length of NICU stay was longer in EONS (24 [15–35] vs. 11 [7–25] days; p=0.016). Lethal cases were observed only in the EONS group (4 cases, 22% vs. 0%; p=0.014). Other perinatal and feeding variables were comparable between groups (all p⩾0.35), with the exception of enteral feeding progression: infants with EONS received lower cumulative enteral volumes during the first week and achieved 150 mL/kg/day later than non-EONS infants (Table 2).

### 3.2. Fecal SCFA Levels Across Postnatal Windows (All Patients)

Table 3 summarizes absolute fecal SCFA concentrations (µmol/g) for all enrolled preterm infants across predefined postnatal windows (TP1–TP5; ∼3, 7, 14, 21, and 28 days of life).

Acetic acid (AA) predominated at all timepoints, showing a steady increase from TP1 (median 360, IQR 222–1530) and TP2 (428, 310–971) toward substantially higher levels by TP3 (4286, 633–4989) and a marked peak at TP4 (21,205, 8670–26,372), followed by a moderate decline at TP5 (9843, 5543–24,117).

Propionic acid (PA) demonstrated a more variable course, with modest levels at TP1–TP2 (17.3 and 10.8 µmol/g) and a pronounced rise by TP3 (34.4, 9.9–67.8), remaining elevated at TP4 (22.2, 20.0–77.5) and stabilizing thereafter (25.2, 14.5–27.1 at TP5).

Butyric acid (BA) exhibited a gradual and comparatively moderate increase (2.4, 2.8, 4.9, 6.2, and 4.3 µmol/g for TP1–TP5, respectively), with the highest interindividual variability observed after the third week of life.

Among minor SCFAs, isobutyric acid (iBA) remained relatively stable during the first two weeks (3.7–5.3 µmol/g at TP1–TP3) and tended to increase thereafter (4.8 at TP4–TP5). Valeric acid (VA) concentrations were low throughout the neonatal period, without a clear monotonic pattern (median range 1.6–3.3 µmol/g).

Isovaleric acid (iVA) and 2-methylbutyric acid (2mBA) were detected at submicromolar to low micromolar levels and displayed slight increases by the end of the first month (TP4–TP5).

In contrast, medium-chain acids hexanoic acid (HA) and octanoic acid (OA) showed higher absolute values and dynamic fluctuations. HA rose from 21.2 µmol/g at TP1 to transient peaks at TP3–TP4 (9.9–13.4), while OA peaked earlier at TP1 (15.7, 6.5–28.1) and gradually declined toward TP5 (6.6, 5.3–9.2).

Overall, total fecal SCFA output increased across the neonatal period, driven primarily by acetic acid and, to a lesser extent, propionic and butyric acids, whereas branched- and medium-chain acids contributed smaller, yet dynamically variable, fractions of the total pool. These aggregate patterns provide a developmental context for group-wise contrasts; comparisons between EONS and non-sepsis infants across the same time windows are presented in the subsequent sections.

Branched-chain acids and valeric acid exhibited low early values with late increases in both medians and dispersion: isobutyrate (iBA) rose from 3.63 (2.66–4.72) at TP1 to 8.59 (3.06–16.02) and 11.02 (3.28–34.58) at TP4–TP5; isovalerate (iVA) from 0.44 (0.14–1.39) at TP1 to 1.85 (0.68–3.80) and 2.91 (0.78–8.65) at TP4–TP5; valerate (VA) from 1.88 (1.43–3.75) to 5.40 (2.26–12.20) and 8.64 (2.62–25.18). Medium-chain acids showed a similar late pattern: hexanoate (HA) and octanoate (OA) remained low through TP1–TP3 and rose at TP4–TP5 (HA to 83.28 and 136.64; OA to 24.69 and 37.73, medians).

Across several analytes, the interquartile ranges and observed ranges widened markedly after TP3 (e.g., AA and PA), indicating increasing between-infant heterogeneity as feeding advances and postnatal adaptation progresses. These distributions provide the reference context for subsequent groupwise and trajectory analyses in this cohort.

### 3.3. Between-Group Differences: EONS vs. Non-Sepsis (Day-Specific Analyses)

**Figure 1:**
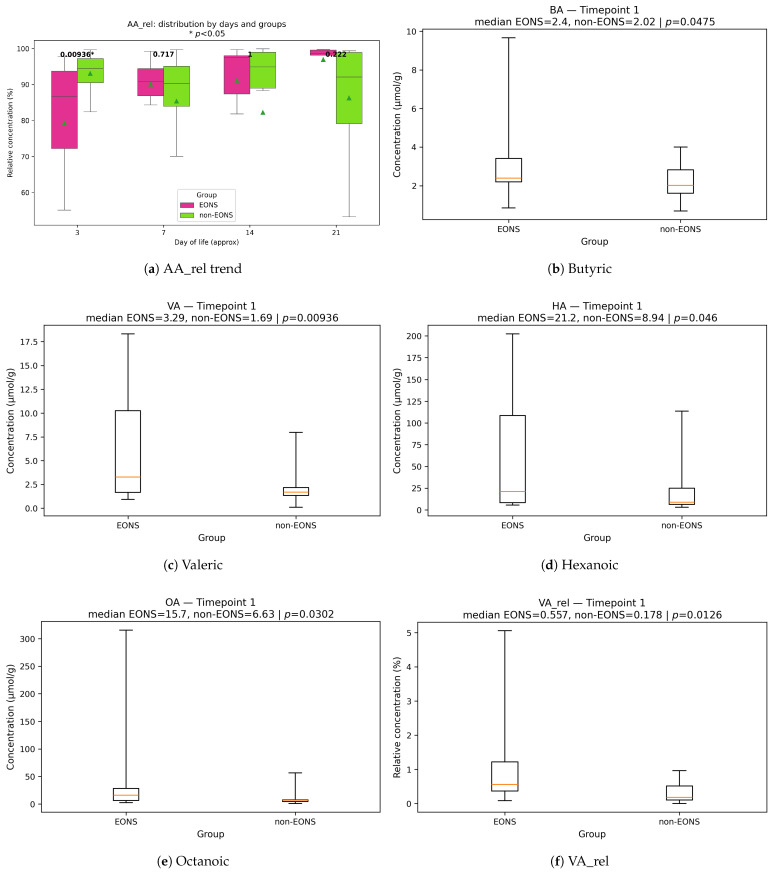

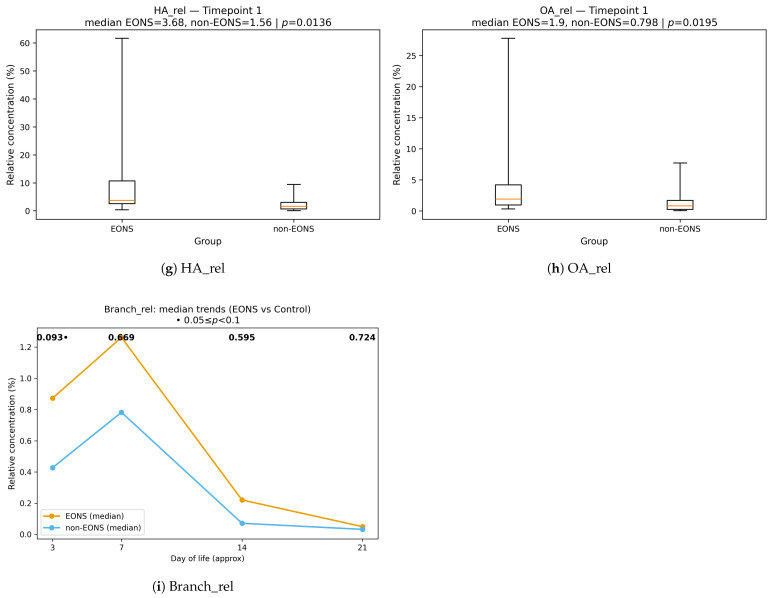
Fecal short-chain fatty acid (SCFA) distribution at the first timepoint (TP1, ∼3 days of life) in infants with early-onset neonatal sepsis (EONS) and non-sepsis controls. (**a**) Temporal trend of relative acetate (AA_rel) concentration across groups (triangle symbols indicate mean values at each timepoint, whereas lines represent group medians across timepoints); (**b**–**h**) boxplots of absolute and relative concentrations for individual acids significantly differing at TP1: butyric (BA), valeric (VA), hexanoic (HA), octanoic (OA), and their relative fractions (VA_rel, HA_rel, OA_rel); (**i**) temporal trend of combined relative contribution of branched SCFAs (Branch_rel). Lines represent group medians across timepoints (orange: EONS; blue: non-EONS). Data are shown as medians with interquartile ranges; *p*-values from Mann–Whitney *U* tests.

**Figure A1:**
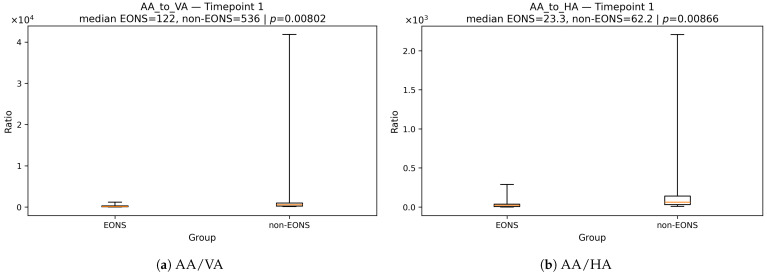

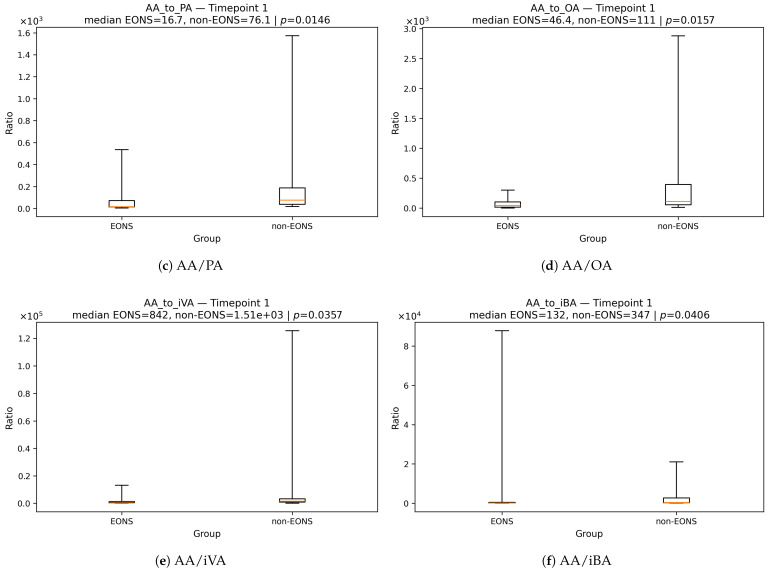
Acetate-to-non-acetate ratios at TP1 (∼3 days of life) in infants with EONS and non-sepsis controls. All ratios (AA/VA, AA/HA, AA/PA, AA/OA, AA/iVA, AA/iBA, and AA/BA) were lower in EONS, indicating a redistribution of the fecal SCFA pool away from acetate and toward branched- and medium-chain acids. Boxplots represent median and interquartile range; *p*-values from Mann–Whitney *U* tests are reported in Table 4.

At TP1 (∼3 DoL), among infants originally enrolled as high risk for NEC but analyzed here for EONS, we observed a pronounced redistribution of the fecal SCFA pool (Table 4). The median relative fraction of acetate (AA_rel) was significantly lower in EONS (86.6 % vs. 94.5 %, p=0.009), consistent with the temporal increase of acetate in EONS versus near-stable levels in non-sepsis infants (trend, Figure 1a). Concordantly, acetate-to-non-acetate ratios were consistently reduced in EONS, indicating a redistribution of the SCFA pool toward non-acetate and branched-chain acids. The strongest differences were observed for AA/VA (121.7 vs. 535.6, p=0.008), AA/HA (23.26 vs. 62.15, p=0.009), AA/PA (16.72 vs. 76.11, p=0.015), AA/OA (46.35 vs. 111.27, p=0.016), AA/iVA (842.45 vs. 1511.37, p=0.036), and AA/iBA (131.64 vs. 347.45, p=0.041), with a similar tendency for AA/BA (p=0.055) (Figure A1a–f).

Non-acetate components predominated in EONS at TP1: butyric (BA, 2.40 vs. 2.02 μM, p=0.047), valeric (VA, 3.29 vs. 1.70 μM, p=0.009), hexanoic (HA, 21.2 vs. 8.94 μM, p=0.046), and octanoic acids (OA, 15.7 vs. 6.64 μM, p=0.030) were elevated, with concordant increases in their relative fractions (VA_rel, p=0.0126; HA_rel, p=0.0136; OA_rel, p=0.0195; Figure 1b–h).

The combined relative contribution of branched SCFAs showed an early elevation in EONS and a decline in statistical significance after week 2 (trend, Figure 1i); at TP1, individual branched fractions demonstrated only tendencies (PA_rel, p=0.055; iBA_rel, p=0.052; iVA_rel, p>0.1) and are therefore not illustrated here (see Table 4).

At TP2 (∼7 DoL), between-group contrasts attenuated: no comparisons reached statistical significance (all p≥0.10). A single borderline trend was observed for the PA/BA ratio, which was lower in EONS (p=0.065; Table 4). The temporal patterns persisted—AA_rel continued to increase in EONS relative to non-sepsis (Figure 1a), and the combined branched fraction remained qualitatively higher without statistical support at this timepoint (Figure 1i). By TP3 (∼14 DoL), selective differences were confined to a single branched-chain ratio: BA/2mBA was higher in EONS (8.90 vs. 4.80; p=0.058), consistent with a transient reweighting among BCFAs rather than a global pool shift; other comparisons, including iBA/2mBA, were not significant (Table 4). By TP4 (∼21 DoL), early-case differences largely attenuated, with a single residual contrast: valeric acid (VA) was lower in EONS (1.88 vs. 3.34 μM; p=0.045). Weak tendencies were also observed for OA/VA (higher in EONS; p=0.065) and for PA-based ratios (PA/iVA and PA/2mBA; both p≈0.093). Overall, EONS-associated alterations appear predominantly *transient*: a broad acetate-to-non-acetate redistribution at TP1, narrowing to a selective BCFA-related ratio by TP3, and minimal residual differences by TP4 (see Table 4).

### 3.4. Multivariate Discrimination at the Earliest Timepoint (PLS–DA at TP1)

**Figure A2:**
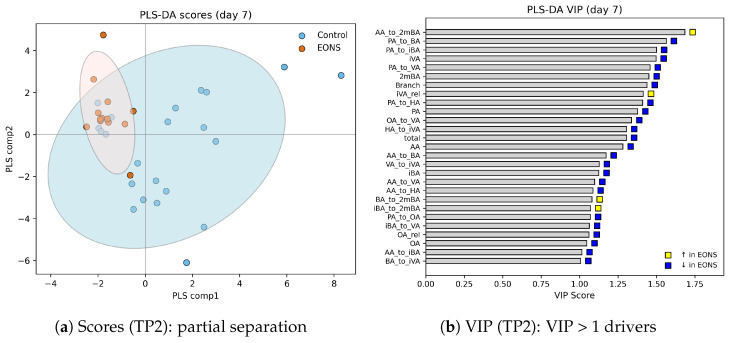

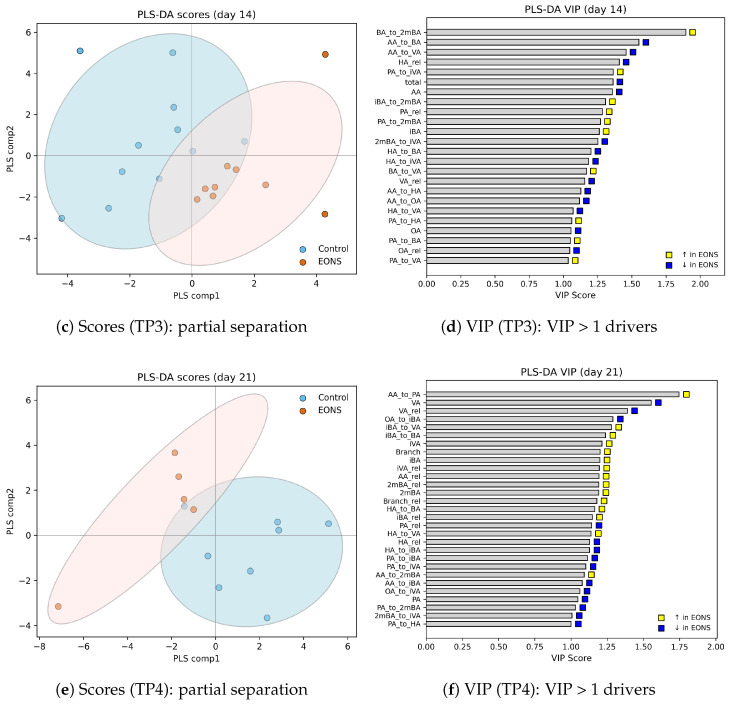
PLS–DA at TP2–TP4 (≈7, 14, 21 DoL). Colored ellipses indicate the 95% confidence regions for each group. Compared with TP1, group separation is weaker or unstable; VIP patterns are attenuated. Yellow = ↑EONS, blue = ↓EONS.

To complement the day-specific tests, we performed timepoint-wise PLS–DA (EONS vs. non-EONS). Because the strongest redistribution of the SCFA pool occurred at the earliest window (TP1, ≈3 DoL), we report TP1 in the main text, whereas PLS–DA panels for TP2–TP4 (scores and VIP) are provided in Appendix A (Figure A2).

**Figure 2:**
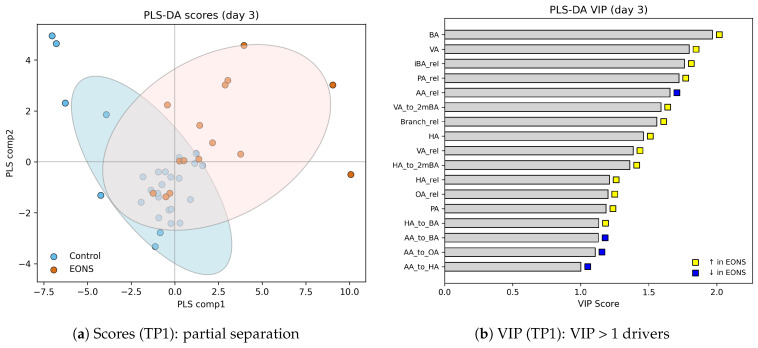
PLS–DA at TP1 (≈3 DoL). (**a**) Scores: partial separation between EONS and non-sepsis; colored ellipses indicate the 95% confidence regions for each group. (**b**) VIP ranking: bars ordered by VIP; yellow = ↑ in EONS, blue = ↓ in EONS. Among the strongest contributors (VIP > 1) are butyric and valeric acids (BA, VA), propionic and branched-chain features (${\mathrm{PA}}_{\mathrm{rel}}$, ${\mathrm{iBA}}_{\mathrm{rel}}$, ${\mathrm{Branch}}_{\mathrm{rel}}$), several VA-related ratios (e.g., VA_to 2mBA_, ${\mathrm{VA}}_{\mathrm{rel}}$), and medium-chain acids (HA), while acetic acid (${\mathrm{AA}}_{\mathrm{rel}}$) contributes in the opposite direction (↓ in EONS).

At TP1, the PLS–DA scores showed a clear partial separation between groups (Figure 2a), in agreement with the univariate contrasts. The VIP ranking (Figure 2b) highlighted multiple non-acetate features among the strongest contributors to discrimination, including butyric and valeric acids (BA, VA), the relative abundance of branched-chain acids (${\mathrm{iBA}}_{\mathrm{rel}}$, ${\mathrm{Branch}}_{\mathrm{rel}}$), propionic and valeric fractions (${\mathrm{PA}}_{\mathrm{rel}}$, ${\mathrm{VA}}_{\mathrm{rel}}$), and several BCFA-related ratios (e.g., VA/2mBA). Acetic acid contributed in the opposite direction, with ${\mathrm{AA}}_{\mathrm{rel}}$ decreased in EONS. Overall, the multivariate pattern at TP1 reflects a coordinated shift away from acetic acid toward propionate-, valerate-, and BCFA-related components of the fecal SCFA pool.

### 3.5. Fecal SCFA Profiles in Older Infants with Confirmed and Suspected NEC

A small auxiliary subgroup of five infants was assessed due to clinical suspicion of necrotizing enterocolitis (NEC). Three of these neonates were the only confirmed NEC cases among all infants admitted to the NICU during the study period (n = 540). One infant demonstrated intestinal bleeding associated with infection during the perinatal period, and one infant had suspected NEC (Bell stage Ib). The clinical characteristics of these five infants are summarized in Table 5. Clinical signs of intestinal pathology in this subgroup appeared between day 4 and day 17 of life.

Fecal samples for SCFA analysis in this subgroup were collected within 24 h after the onset of intestinal symptoms in all five infants. Thus, the SCFA measurements reflect the early phase of NEC suspicion or perinatal intestinal infection rather than a baseline, asymptomatic state. At the time of sampling, these infants were more mature than the main cohort in terms of gestational age (median 36.6 weeks [36.0; 37.0]). Enteral feeding was discontinued at the onset of symptoms; therefore, the feeding type and volume indicated in Table 5 refer to the last 24 h of enteral nutrition prior to clinical deterioration (i.e., the day preceding stool collection). Their fecal SCFA concentrations (Table 6) demonstrated substantial heterogeneity across all measured acids. Acetic acid (AA) reached high values (median 7369 µmol/g), while propionic acid (PA) showed wide interindividual variation (19.7–4111.2 µmol/g). Branched-chain fatty acids—including isobutyric (iBA), isovaleric (iVA), and 2-methylbutyric acid (2mBA)—were consistently detectable, and medium-chain acids such as hexanoic (HA) and octanoic acid (OA) exhibited the highest variability, particularly in infants with perinatal intestinal infection or suspected NEC. In descriptive terms, infants with confirmed NEC (Bell IIa–IIb) tended to have lower PA and branched-chain SCFA levels, as well as lower HA and OA concentrations, whereas the highest PA, branched-chain SCFA, and MCFA values were observed in the subgroup with perinatal infection-related intestinal symptoms and suspected NEC.

These descriptive findings illustrate the diversity of fecal SCFA profiles in infants evaluated for suspected NEC and highlight the potential metabolic signatures that may characterize this clinical setting. Although the sample size is insufficient for formal statistical comparisons, the observed differences between confirmed NEC, perinatal intestinal infection, and suspected NEC suggest that targeted SCFA profiling may help refine early metabolic phenotyping in future NEC-focused cohorts. The results support the methodological framework used in the present study and emphasize the importance of integrating SCFA measurements into prospective investigations of NEC risk, early diagnosis, and pathophysiology.

### 3.6. Discussion

In this longitudinal cohort study, we identified distinct temporal shifts in fecal short-chain fatty acids in preterm infants who developed early-onset neonatal sepsis (EONS). Although SCFAs are increasingly recognized as key microbial metabolites involved in epithelial maturation, immune modulation, and inflammatory signaling [14,15,16,21,22,23,24], their dynamic behavior during systemic infection has remained largely unexplored. Recent reviews highlight the potential importance of SCFAs in neonatal sepsis [11,12,13], but existing evidence relies mostly on single-timepoint measurements or on systemic rather than intestinal readouts. To our knowledge, this is the first study to characterize time-resolved fecal SCFA and BCFA trajectories during EONS across the first month of life.

A consistent finding was the early reduction in the relative dominance of acetic acid, accompanied by higher proportions of non-acetate acids—including propionic, valeric, and branched-chain fatty acids—at the initial timepoint. Such deviations suggest that EONS may disrupt microbial metabolic output from the earliest days of life. Reduced acetate dominance may reflect delayed establishment of key saccharolytic commensals or impaired epithelial utilization of acetate during systemic inflammation, both of which have been reported in preterm infants with inflammatory morbidities [7,27,28,29,30]. Conversely, the relative enrichment of non-acetate SCFAs and BCFAs may reflect early perturbations in fermentation pathways or disruption of normal ecological succession in the immature gut [8,10].

The pronounced differences observed in acetate-to-non-acetate ratios provide additional evidence of metabolic imbalance. Ratio-based measures offer an internally normalized view of fermentation activity and are robust against the large inter-individual variation in total SCFA output typical in preterm infants, where feeding regimens, transit time, and microbial maturity vary considerably [45,46]. Previous studies on SCFAs in NEC and sepsis [20,32,47,48] have reported inconsistent absolute concentrations, underscoring the limitations of single-point measurements and supporting the value of longitudinal, ratio-informed approaches.

An additional consideration is that we did not adjust for the absolute volume of enteral substrate at each timepoint, which may influence the availability of medium-chain triglycerides and, consequently, the fecal levels of medium-chain fatty acids (MCFAs). Prior neonatal nutrition studies demonstrate that dietary fat quantity and formulation—particularly the proportion of medium-chain triglycerides—can substantially affect MCFA absorption and downstream fecal levels [31,49,50,51]. Although our time-structured design provides internally consistent trajectories, future work should explore feeding-volume normalization to refine interpretation of MCFA-related dynamics. Detailed, day-by-day quantitative data on enteral feeding volume and its progression were not available for all infants in this pilot cohort, although summary indicators such as total enteral intake during the first week and time to reach 150 mL/kg were available and are reported in Table 2. These aggregated values, however, do not allow precise assessment of how substrate load varied across the entire neonatal period and may therefore contribute to variability in absolute SCFA levels. Future studies should incorporate standardized nutritional data, including feeding-volume trajectories, to refine interpretation of fermentation-related metabolic patterns. Nevertheless, the longitudinal, repeated-measures design of this study provides substantially greater internal consistency and interpretive reliability than cross-sectional sampling, thereby strengthening the robustness of the observed SCFA trajectories despite this limitation.

These findings align with emerging evidence linking intestinal immaturity, dysbiosis, and systemic inflammation in preterm infants. Neonatal sepsis has been repeatedly associated with delayed colonization by beneficial anaerobes, instability of microbial communities, and enrichment of facultative or opportunistic taxa [7,8,36]. Because SCFAs modulate epithelial energy supply, tight-junction expression, mucus production, and innate immune responses [21,22,23,24], the alterations documented here likely represent both a marker and a mediator of early gut–systemic inflammatory crosstalk.

Although NEC and EONS are distinct clinical entities, early-stage NEC-risk phenotypes (Bell stage I) also demonstrate metabolic and microbial disturbances before overt intestinal injury [17,19]. Some of these early deviations—such as altered acetate dominance or increased variability in non-acetate fractions—may reflect a broader inflammatory or maturational vulnerability in the preterm gut [20]. Our findings extend this concept to the setting of systemic infection, indicating that EONS is accompanied by reproducible SCFA shifts that may represent a condition-specific metabolic signature rather than a generic consequence of illness.

The principal strengths of this study include its longitudinal design and high-resolution sampling across predefined postnatal windows. Although a handful of preliminary studies have explored gut-microbial metabolites in preterm infants during the first month of life [9,52,53], time-resolved, fecal SCFA trajectories in the context of systemic neonatal infection remain almost entirely uncharted. This serial approach captures within-infant evolution and reveals structured metabolic patterns that would be missed by single-timepoint sampling.

Because clinical recognition of EONS and the initiation of empiric ampicillin–gentamicin therapy both occurred within the first hours of life, TP1 stool samples were collected after treatment had begun. Antibiotic exposure may also have occurred in some infants without EONS, as empiric therapy is commonly initiated in very preterm neonates during early evaluation [37,54]. Indeed, most newborns in the non-EONS group (27/31) received empirical antibiotics beginning on the first day of life; in 10 of these infants treatment was discontinued after 48 h once EONS or pneumonia had been excluded. However, the specific pattern of SCFA alterations observed in the EONS group—most notably the loss of acetate dominance and the relative enrichment of branched-chain and other non-acetate acids—aligns more closely with metabolic responses to systemic inflammation than with antibiotic exposure alone [20,46,47,48,55]. These shifts resemble previously described signatures of host–microbial metabolic stress and microbiome–metabolome perturbation in preterm infants with systemic infection or intensive-care-related exposures [8,10,13,52]. Nevertheless, because infection and empiric treatment partly overlap in timing, their individual contributions cannot be fully separated within this design. Future studies with pre-symptomatic sampling or stratified antibiotic exposure will be required to disentangle these effects.

Limitations include the modest cohort size, which reduces statistical power. As this was an exploratory pilot cohort, no a priori sample size calculation was performed, and the study was not powered to investigate NEC-specific outcomes; therefore, all NEC-related observations should be regarded as descriptive and hypothesis-generating. Additional limitations include the lack of microbiome or immune profiling, which precludes direct mechanistic mapping. Clinical factors such as feeding modality, antibiotic exposure, and respiratory support may have contributed to the observed variability. In addition, infants in the EONS group exhibited overall greater illness severity, including lower birth weight, lower Apgar scores, a higher frequency of respiratory support, and increased mortality. These baseline differences reflect the clinical burden of early-onset sepsis but also represent potential confounding factors when interpreting metabolic readouts. To mitigate the influence of global disease severity and variability in stool mass or transit time, our analyses focused primarily on ratio-based SCFA metrics, which provide internally normalized measures of fermentation activity. Such ratios better capture shifts in microbial metabolic patterns specific to EONS rather than nonspecific consequences of severe systemic illness. To further assess whether the TP1 metabolic differences could be explained solely by greater illness severity, we performed a targeted sensitivity analysis excluding infants with fatal outcomes or prolonged invasive ventilation. The characteristic reduction in acetate dominance and the relative enrichment of non-acetate SCFAs in the EONS group remained evident, indicating that these early metabolic alterations are unlikely to be driven solely by severity-related factors. Nevertheless, future adequately powered multicenter cohorts will be required to enable formal adjusted modeling and to disentangle disease-specific metabolic signatures from the broader effects of severe neonatal illness. Larger, multicenter cohorts integrating SCFA measurements with metagenomic and immunologic readouts will be essential to determine whether these metabolic trajectories represent causal contributors to EONS vulnerability, compensatory responses, or epiphenomena of severe illness [7,8].

Overall, this study provides the first longitudinal evidence that EONS in preterm infants is accompanied by structured changes in the fecal SCFA landscape. The early reduction in acetic-acid dominance and the enrichment of non-acetate components highlight the gut’s metabolic response as a potential early indicator of systemic inflammatory stress. These findings lay the groundwork for future research on gut–systemic communication in neonatal sepsis and support the development of metabolically informed approaches to early risk assessment in high-risk neonatal populations.

## 4. Conclusions

This study provides the first longitudinal characterization of fecal short-chain fatty acid dynamics in preterm infants with early-onset neonatal sepsis (EONS). We demonstrate that EONS is accompanied by reproducible, time-structured alterations in intestinal fermentation, including an early reduction in acetic-acid dominance and a relative enrichment of non-acetate components. These shifts are evident from the initial days of life and are most clearly captured through ratio-based metrics, which offer an internally normalized view of microbial metabolic function and are more robust than absolute concentrations in the highly variable preterm setting.

The metabolic signature identified here suggests that the intestinal microbiome of infants with EONS undergoes early functional reorganization during systemic infection, supporting growing evidence that gut–systemic inflammatory crosstalk is highly active in the neonatal period. Although NEC and EONS represent distinct clinical entities, some early deviations observed in NEC-risk phenotypes parallel the initial SCFA imbalance seen in EONS, indicating potential points of convergence in inflammatory vulnerability.

Our findings highlight fecal SCFAs as a promising non-invasive marker of early systemic infection in preterm infants. The serial sampling approach used in this study captures within-infant trajectories that are not accessible through single-timepoint measurements and may provide a basis for developing metabolically informed risk stratification tools. Future work should validate these observations in larger, multicenter cohorts and integrate SCFA profiling with microbiome and immune analyses to elucidate mechanistic pathways and identify actionable targets for early intervention.

## Figures and Tables

**Table 1:** Calibration parameters and linearity of the GC–MS method.

Compound	RT (min)	Range (μM)	*m*/*z*	LOD/LOQ (μM)	Calibration Equation	$R^{2}$
Internal standards						
d_4_-AA	5.21	50	63	—	—	—
^13^C_2_-BA	6.36	5	62	—	—	—
Straight-chain SCFAs						
AA	5.23	2–160	60	0.3/1.0	y=0.017690x−0.201383	0.9974
PA	5.78	0.2–16	74	0.04/0.1	y=0.053251x+0.019401	0.9911
BA	6.36	0.2–16	60	0.05/0.2	y=0.250122x+0.040709	0.9980
VA	7.02	0.02–1.6	60	0.004/0.01	y=0.369129x+0.026151	0.9931
CA	7.62	0.02–1.6	60	0.004/0.01	y=0.403215x+0.111489	0.8989
OA	8.73	0.02–1.6	60	0.005/0.02	y=0.400269x+0.143028	0.9627
Branched-chain and methyl-substituted acids						
iBA	5.70	0.2–16	43	0.05/0.2	y=0.179669x−0.037038	0.9966
iVA	6.62	0.02–1.6	60	0.004/0.01	y=0.398671x+0.016952	0.9957
2mBA	6.62	0.02–1.6	74	0.004/0.01	y=0.313886x	0.9971
2mVA	7.18	0.02–1.6	74	0.005/0.02	y=0.574202x	0.9947
3mVA	7.33	0.02–1.6	60	0.004/0.01	y=0.404234x+0.012466	0.9957

Note. RT = retention time; ISTD = internal standard; $R^{2}$ = coefficient of determination; *m*/*z* identification ion. LOD = 3.3 × SD/b; LOQ = 3 × LOD. Internal standards: d_4_-AA (10^3^ µM) and ^13^C_2_-BA (10^2^ µM), 5 µL added per sample.

**Table 2:** Patients’ characteristics.

Characteristic	Total (N = 49)	EONS (N = 18)	Without EONS (N = 31)	*p*-Value
Gestational age, weeks	31 (28.5–32)	30 (26.5–31.6)	31.2 (29.3–32)	0.10
Birth weight, g	1250 (998–1615)	1121 (840–1329)	1440 (1092–1679)	0.049
Male sex, n (%)	22 (45%)	10 (56%)	13 (42%)	0.390
Apgar score, 1st minute	6 (5–7)	5 (4–6)	6 (6–7)	<0.001
Apgar score, 5th minute	7 (6–8)	6 (6–7)	8 (7–8)	0.03
Cesarean section, n (%)	47 (96%)	18 (100%)	29 (94%)	0.526
RDS steroid prophylaxis, n (%)	32 (65%)	10	22 (71%)	0.355
Multiples, n (%)	22 (45%)	8	14 (45%)	1.000
Surfactant treatment, n (%)	34 (69%)	18	16 (52%)	<0.001
Invasive mechanical ventilation, n (%)	34 (69%)	17	15 (48%)	0.001
EONS clinical, n (%)	17 (35%)	17	NA	—
EONS culture proven, n (%)	1 (2%)	1	NA	—
Antibacterial therapy (empirical, during initial EONS evaluation), n (%)	10 (20%)	0	10 (32%)	0.008
Antibacterial therapy (full course), n (%)	35 (71%)	18 (100%)	17 (55%)	<0.001
Number of antibacterial therapy courses	2 (1–3)	3 (2–3)	1 (1–2)	<0.01
Enteral substrate exclusively MOM, n (%)	16 (33%)	6	10 (32%)	1.000
Mixed enteral feeding, n (%)	32 (65%)	11	21 (68%)	0.758
Volume of enteral feeds during the first week (mL/kg)	212 (127–485)	170 (141–298)	245 (126–486)	0.037
Time to enteral volume 150 mL/kg (days)	11 (7–14)	10 (11–19)	15 (7–13)	0.002
Nil per os, n (%)	1 (2%)	1	0	0.367
Length NICU stay, days	16 (10–27)	24 (15–35)	11 (7–25)	0.016
Mortality, n (%)	4 (8%)	4 (22%)	0	0.014

Continuous data are presented as Me (Q1–Q3) and were compared using the Mann–Whitney U test; categorical data are shown as n (%) and were compared using Fisher’s exact test. MOM—mother’s own milk.

**Table 3:** Absolute fecal SCFA concentrations across postnatal windows (all patients; no group stratification).

SCFA ( µmol/g)	TP1 (≈3 DoL, n = 25)	TP2 (≈7 DoL, n = 22)	TP3 (≈14 DoL, n = 17)	TP4 (≈21 DoL, n = 15)	TP5 (≈28 DoL, n = 14)
Acetic (AA)	715 [222; 1530] (74; 4706)	633 [310; 971] (37; 10,039)	2120 [633; 4990] (212; 28,159)	1425 [1242; 26,372] (1425; 35,186)	1243 [941; 24,117] (1243; 38,390)
Butyric (BA)	2.83 [2.21; 3.41] (0.85; 9.66)	2.98 [2.08; 3.53] (0.86; 11.31)	2.83 [2.28; 5.72] (2.83; 17.42)	3.62 [1.77; 6.72] (1.31; 22.76)	2.62 [2.00; 5.99] (2.62; 7.67)
Propionic (PA)	10.64 [9.59; 26.53] (2.24; 84.84)	14.64 [6.35; 27.25] (0.74; 77.24)	11.64 [6.35; 83.04] (6.34; 691.04)	22.64 [9.24; 597.04] (9.24; 597.04)	3.74 [2.24; 28.94] (3.74; 28.94)
Isobutyric (iBA)	3.37 [2.49; 4.28] (0.04; 16.89)	3.62 [2.73; 3.99] (0.04; 10.94)	3.61 [2.70; 7.91] (0.04; 23.30)	3.72 [3.06; 7.86] (3.06; 50.21)	3.26 [3.23; 4.75] (3.26; 4.76)
Valeric (VA)	1.88 [1.66; 10.26] (0.92; 18.32)	1.66 [1.34; 2.75] (1.07; 3.49)	2.11 [1.90; 2.65] (1.25; 5.07)	1.43 [1.96; 2.06] (1.43; 3.03)	1.84 [2.12; 2.50] (1.83; 2.60)
Isovaleric (iVA)	0.44 [0.34; 1.39] (0.04; 4.44)	0.60 [0.26; 0.74] (0.04; 16.54)	0.53 [0.22; 1.04] (0.14; 13.34)	0.34 [0.58; 4.44] (0.34; 48.24)	0.34 [0.42; 1.19] (0.34; 1.34)
2-Methylbutyric (2mBA)	0.64 [0.41; 1.00] (0.27; 2.26)	0.44 [0.34; 0.59] (0.18; 11.93)	0.59 [0.34; 0.97] (0.29; 6.23)	1.41 [0.45; 5.51] (0.39; 46.69)	0.68 [0.52; 1.56] (0.37; 2.43)
Hexanoic (HA)	21.23 [8.24; 108.69] (5.63; 202.34)	7.83 [5.93; 13.64] (5.03; 30.24)	7.44 [5.03; 17.04] (3.44; 28.33)	9.04 [10.13; 20.54] (9.04; 22.34)	10.34 [9.64; 11.98] (8.94; 13.63)
Octanoic (OA)	15.73 [6.49; 28.09] (2.24; 315.64)	5.83 [4.33; 8.24] (3.04; 28.24)	5.63 [3.47; 8.44] (2.44; 167.94)	7.24 [10.13; 15.13] (7.24; 19.14)	6.63 [5.33; 9.23] (4.04; 11.84)

Values are median [Q1; Q3] (min; max). Units: µmol/g. DoL: day of life.

**Table 4:** Day-specific EONS vs. non-sepsis comparisons (Mann–Whitney *p*-values). Bold: p<0.05; italics: 0.05≤p<0.10.

Metric	TP1 (≈3 DoL)	TP2 (≈7 DoL)	TP3 (≈14 DoL)	TP4 (≈21 DoL)
${\mathrm{AA}}_{\mathrm{rel}}$	**0.009**	0.717	1.000	0.222
AA/BA	*0.055*	0.737	0.595	0.222
AA/HA	**0.009**	0.737	0.595	0.222
AA/iBA	**0.041**	0.717	0.543	0.943
AA/iVA	**0.036**	0.889	0.595	0.435
AA/OA	**0.016**	0.843	0.704	0.943
AA/PA	**0.015**	0.576	0.879	0.222
AA/VA	**0.008**	0.429	0.820	0.435
BA	**0.047**	0.987	0.224	0.524
BA/2mBA	0.813	0.167	*0.058*	0.524
${\mathrm{Branch}}_{\mathrm{rel}}$	0.093	0.132	0.109	0.435
HA	**0.046**	0.364	0.220	0.438
${\mathrm{HA}}_{\mathrm{rel}}$	**0.014**	0.531	0.820	0.724
HA/2mBA	*0.055*	0.372	0.879	0.943
${\mathrm{iBA}}_{\mathrm{rel}}$	*0.052*	0.717	0.543	0.524
OA	**0.030**	0.256	1.000	0.558
${\mathrm{OA}}_{\mathrm{rel}}$	**0.019**	0.818	0.649	0.943
OA/2mBA	**0.046**	0.392	0.879	0.943
OA/VA	0.834	0.323	0.197	*0.065*
${\mathrm{PA}}_{\mathrm{rel}}$	*0.055*	0.645	0.595	0.222
PA/BA	0.651	*0.065*	0.612	0.524
PA/iVA	0.262	0.748	0.093	0.820
VA	**0.009**	1.000	0.171	**0.045**
${\mathrm{VA}}_{\mathrm{rel}}$	**0.013**	0.340	0.820	0.524
VA/2mBA	**0.022**	0.122	0.287	0.435

Mann–Whitney *U* tests comparing EONS vs. non-sepsis; no multiplicity adjustment applied in this screening table.

**Table 5:** Clinical characteristics of older preterm infants evaluated for NEC (Bell stage I–II).

Case	GA (weeks)	BW (g)	Delivery Mode	Sex	Apgar 1st min	Apgar 5th min	Feeding Type and Volume	Congenital Heart Disease	Clinical Signs Onset (DoL)	Diagnosis
Case 1 (U)	34.5	2549	CS	male	7	8	Mixed, 120 mL/kg/day	Yes (ventricular septal defect)	7	NEC 2b ^a^
Case 2 (S)	36.6	2637	CS	male	7	8	Mixed, 145 mL/kg/day	Yes (Tetralogy of Fallot)	10	NEC 2a ^b^
Case 3 (Kal)	38.0	2940	CS	male	7	8	Formula 150 mL/kg/day	No	4	NEC 2a ^c^
Case 4 (Kar)	36.0	2745	CS	female	7	9	Formula 155 mL/kg/day	No	5	Feeding intolerance due to infection
Case 5 (F)	37.0	3198	CS	female	8	9	Formula 160 mL/kg/day	No	17	NEC 1b (suspected)

CS—cesarean section; DoL—day of life; mixed feeding—mother’s own milk + formula. ^a^ Stool culture: *Escherichia coli* 10^6^ CFU/g on day 3 of life. ^b^ Pharyngeal swab: *Klebsiella pneumoniae* 10^4^ CFU/mL; stool: *Klebsiella pneumoniae* 10^7^ CFU/g. ^c^ Pharyngeal swab and stool (day 4): *Enterobacter cloacae* and *Pseudomonas aeruginosa*.

**Table 6:** Summary of fecal SCFA profiles in older gestational age infants evaluated for NEC. Values are median [min; max].

SCFA (µmol/g)	Total (Cases 1–5, n=5)	NEC Bell IIa–IIb (Cases 1–3, n=3)	Perinatal Intestinal Infection/Suspected NEC (Cases 4–5, n=2)
**Absolute Concentrations (µmol/g)**			
Acetic acid (AA)	7369.0 [805.0; 18,128.0]	7369.0 [4523.0; 18,128.0]	6886.5 [805.0; 12,968.0]
Propionic acid (PA)	104.7 [19.7; 4111.2]	104.7 [19.7; 1581.8]	2098.3 [85.4; 4111.2]
Butyric acid (BA)	3.79 [2.89; 8.58]	3.39 [2.89; 6.49]	6.19 [3.79; 8.58]
Branched-chain SCFAs ^a^	17.01 [7.73; 19.61]	11.61 [7.73; 17.01]	19.20 [18.78; 19.61]
Valeric acid (VA)	2.81 [1.13; 4.85]	2.61 [1.13; 4.85]	4.81 [4.77; 4.85]
Hexanoic acid (HA)	13.9 [3.8; 44.8]	9.8 [3.8; 13.9]	35.25 [25.7; 44.8]
Octanoic acid (OA)	7.6 [2.4; 30.8]	7.6 [2.4; 7.6]	23.4 [16.0; 30.8]

^a^ Sum of isobutyric, isovaleric, and 2-methylbutyric acids.

## Data Availability

The datasets generated and analyzed during the current study are not publicly available due to ethical and privacy restrictions related to clinical data from preterm neonates. De-identified data may be made available from the corresponding author upon reasonable request and subject to institutional approval.

## Abbreviations

The following abbreviations are used in this manuscript:

EONS	Early-onset neonatal sepsis
NEC	Necrotizing enterocolitis
NICU	Neonatal intensive care unit
GA	Gestational age
DoL	Day of life
TP	Timepoint
SCFA	Short-chain fatty acid
BCFA	Branched-chain fatty acid
MCFA	Medium-chain fatty acid
MCT	Medium-chain triglyceride
GC–MS	Gas chromatography–mass spectrometry
PLS–DA	Partial least squares–discriminant analysis
LOD	Limit of detection
LOQ	Limit of quantification
SIM	Selected ion monitoring
IQR	Interquartile range
AA	Acetic acid
PA	Propionic acid
BA	Butyric acid
VA	Valeric acid
iBA	Isobutyric acid
iVA	Isovaleric acid
2mBA	2-methylbutyric acid
${\mathrm{Branch}}_{\mathrm{rel}}$	Combined relative fraction of branched-chain fatty acids (iBA, iVA, 2mBA, etc.)
HA	Hexanoic acid
OA	Octanoic acid
